# Supplementary material for: Genetic and Antiviral Potential Characterization of Four Insect-Specific Viruses Identified and Isolated from Mosquitoes in Yunnan Province
Source: Viruses. 2025 Apr 23;17(5):596. doi: 10.3390/v17050596 (PMC12116109; doi:10.3390/v17050596)
Supplement: Supplementary file 1 [file viruses-17-00596-s001.zip › Table S1.pdf]

- The primers and PCR product lengths for the TANAV 、 CxFV 、 AeFV and LTNV.(CxFV/AeFV/TANAV/LTNV newly designed)

name	Gene	Name of primer	Sequence (5’ – 3’)	Product Size (bp)
Tanay virus (TANAV)	RdRp	TANAV-F	ATAATGAACCKTTTKCCGAYGAYTC	1605bp
		TANAV-R	TCMGGAGCDGCRATCATRTGAGC	
Culex orthoflavivirus (CxFV)	NS5	CxFV-F	ACTGGTGACGTTCAAGGCCATAA	1551bp
		CxFV-R	GCCGTGATCAGGTGCTGGTCATC	
Aedes orthoflavivirus (AeFV)	NS5	AeFV-F	CGCGATTTAAGACTAGGGTTTGCTGC	200bp
		AeFV-R	TCGGGTCCTTTACTCTGATCCACGGG	
La Tina virus (LTNV)	NS5	LTNV-F	GGTAACATACGCCCTGAATACG	110bp
		LTNV-R	TCGCTCTTTAGATCCAATTAAG	
