# Supplementary material for: Genetic and Antiviral Potential Characterization of Four Insect-Specific Viruses Identified and Isolated from Mosquitoes in Yunnan Province
Source: Viruses. 2025 Apr 23;17(5):596. doi: 10.3390/v17050596 (PMC12116109; doi:10.3390/v17050596)
Supplement: Supplementary file 1 [file viruses-17-00596-s001.zip › Table S2.pdf]

The GenBank accession numbers of the strains and sample information.

(Sequences represent viral isolates, not PCR products from pooled samples.)

Virus Family	Virus	Strain number	GenBank accession numbers	species
unclassified viruses	Tanay virus(TANAV)	NBHKW	PV165524	*Culex tritaeniorhynchus Giles*
Flaviviridae	Culex flavivirus(CxFV)	PEKW	PV165384	*Culex tritaeniorhynchus Giles*
Flaviviridae	Aedes flavivirus(AEFV)	JH2YW	PV165522	*Aedes albopictus*
Flaviviridae	La Tina virus	LHYW	PV165523	*Aedes albopictus*
Flaviviridae	DENV-2	-	KM204118.1	*Aedes albopictus*
