# Supplementary material for: Genetic and Antiviral Potential Characterization of Four Insect-Specific Viruses Identified and Isolated from Mosquitoes in Yunnan Province
Source: Viruses. 2025 Apr 23;17(5):596. doi: 10.3390/v17050596 (PMC12116109; doi:10.3390/v17050596)
Supplement: Supplementary file 1 [file viruses-17-00596-s001.zip › Table S3.pdf]

Fluorescent Quantitative Primer Probe Information.

Virus	Primer and probe sequences	Tm value
TANAV	ATGGGACCGTTAYATCGCCAAAG<br>AGCAAGTGTTCCGAGGTATTATCAGG<br>(FAM)TTGAGTACCCCTTGTCCGGCRTTG(BHQ1)	58℃
LTNV	CGCGATTTAAGACTAGGGTTTGCTGC<br>TCGGGTCCTTTACTCTGATCCACGGG<br>(FAM)TCCGCCGTYCCHTCAAACTGGTTC(BHQ1)	60℃
CxFV	TTGTTGTGCGTGGCTATWGCGAAG<br>TGTCTGCARCATGGTCATCTTTTCC<br>(FAM)GRTGGCCGATGTGGGTGGTGATAC(BHQ1)	60℃
AeFV	TCGCCAAAGAACATGGTTTTGATTG<br>AAGTGTTCCGAGGTATTATCAGGTG<br>(FAM)CCCAAATTGAGTACYCCTTGTCCSGC(BHQ1)	60℃
DENV-2	CAAMCYRTGGAAGCTGTACGCA<br>CGTTCTGTGCCTGGAATGATG<br>(FAM)AACAGCATATTGACGCTGGGARAGACC(BHQ1)	60℃
