# Supplementary material for: Genetic and Antiviral Potential Characterization of Four Insect-Specific Viruses Identified and Isolated from Mosquitoes in Yunnan Province
Source: Viruses. 2025 Apr 23;17(5):596. doi: 10.3390/v17050596 (PMC12116109; doi:10.3390/v17050596)
Supplement: Supplementary file 1 [file viruses-17-00596-s001.zip › Table S4.pdf]

Information on primers used to amplify the full length of Tanay virus.

No.		Name	Sequence (5'—3')
1	Round1	TANV-WF-1	ATGTAGGTWAGCTTTGAGTAATAATC
2		TANV-R-1	CAATAAAACTRCTTGCTATTACTGCC
3	Round2	TANV-NF-1	ATGTAGGTWAGCTTTGAGTAATAATC
4		TANV-NR-1	TTTCRACTATAGTACCTTTAGCTTG
5	Round1	TANV-WR-2	CAGGAGGATTCAARTTACGTTTCTG
6		TANV-WF-2	CATGGMAAGACTCGTGAAATWGTYG
7	Round2	TANV-NR-2	CAGGAGGATTCAARTTACGTTTCTG
8		TANV-NF-2	GTCYCAAGTTRATGTTGCTAAMTTG
9	Round1	TANV-WF-3	CAGCTCGTTCATGGTTTTATG
10		TANV-WR-3	TKCCAATTGGTAAAYAAATAAATAAAG
11	Round2	TANV-NF-3	CAGCTCGTTCATGGTTTTATG
12		TANV-NR-3	CRAATTTTAGGAKTTTCAACCCAGGG
13	Round1	TANV-WF-4	TATGGGATTCRACWATTTCACGAGTC
14		TANV-WR-4	GGTAAAGTTTGTGARTTTTCGGC
15	Round2	TANV-NF-4	TATGGGATTCRACWATTTCACGAGTC
16		TANV-NR-4	TATTGTCGCATGGTAAAGTTTG
17	Round1	TANV-WR-5	GATTTAAAGACCACATTACCACCATC
18		TANV-WF-5	TACCATTCTAAAACCCGCACGTAC
19	Round2	TANV-NF-5	AGCATGGCGTCTACTATGGC
20		TANV-NR-5	GATTTAAAGACCACATTACCACCATC
21	Round1	TANV-WR-6	CACCAATTCATGATAATTATACTTAC
22		TANV-WF-6	ATATATCGTTRAYTTTGTTTGGTKTCTG
23	Round2	TANV-NR-6	CACCAATTCATGATAATTATACTTAC
24		TANV-NF-6	TTCCTCGYGTGCTCTACAAC
25	Round11	TANV-WF-7	TCMGGAGCDGCRATCATRTGAGC
26		TANV-WR-7	ATAATGAACCKTTTKCCGAYGAYTC
27	Round2	TANV-NF-7	GCWAACACTATCGARACDAAAG
28		TANV-NR-7	TACTCAAAGCTWACYTACATTTCYG
29	Round1	TANV-WF-8	AGAGCCTGACGCCTATTCTCATT

No.		Name	Sequence (5’—3’)
30		TANV-WR-8	GGAWCCTTGGAARGCHGC TATCG
31	Round2	TANV-NF-8	AGAGCCTGACGCCTATTCT CATT
32		TANV-NR-8	TGCCGCTACATCCATAGGT ATTC
33	Round1	TANV-WR-9	AATGAGAATAGGCGTCAGG CTC
34		TANV-WF-9	TRAAYGATGGTGGTAATGT RGTC
35	Round1	TANV-NR-9	AATGAGAATAGGCGTCAGG CTC
36		TANV-NF-9	TTTACTTTCCAYGATAAYG CYG
